# Birt-Hogg-Dubé syndrome in apparent primary spontaneous pneumothorax patients; results and recommendations for clinical practice

**DOI:** 10.1186/s12890-022-02107-7

**Published:** 2022-08-26

**Authors:** Jincey D. Sriram, Irma van de Beek, Paul C. Johannesma, Michiel H. van Werkum, Tijmen J. W. T. van der Wel, Elise M. Wessels, Hans J. J. P. Gille, Arjan C. Houweling, Pieter E. Postmus, Hans J. M. Smit

**Affiliations:** 1 Department of Pulmonology, Rijnstate Hospital Arnhem, Wagnerlaan 55, 6815 AD Arnhem, The Netherlands; 2 Department of Human Genetics, Amsterdam UMC, Vrije Universiteit Amsterdam, De Boelelaan 1118, 1081 HZ Amsterdam, The Netherlands; 3 Department of Pulmonology, Amsterdam UMC, Vrije Universiteit Amsterdam, De Boelelaan 1118, 1081 HZ Amsterdam, The Netherlands; 4 Department of Radiology, Rijnstate Hospital, Wagnerlaan 55, 6815 AD Arnhem, The Netherlands; 5 Department of Pulmonology, Leiden University Medical Center, Albinusdreef 2, 2333 ZA Leiden, The Netherlands; 6 Department of Surgery, Gelderse Vallei Ziekenhuis, Willy Brandtlaan 10, 6716 RP Ede, The Netherlands

**Keywords:** BHD, Birt-Hogg-Dubé syndrome, Pneumothorax, Primary spontaneous pneumothorax, PSP

## Abstract

**Background:**

Birt-Hogg-Dubé syndrome (BHD) is an inherited disease caused by pathogenic variants in the *FLCN* gene. One of the characteristics is the increased risk for spontaneous pneumothorax, likely due to the presence of pulmonary cysts mainly distributed under the carina. Due to variable expression and lack of awareness, BHD is likely to be underdiagnosed*.* We aimed to examine the prevalence of BHD in patients presenting with an apparent primary spontaneous pneumothorax and to evaluate the contribution of chest CT in establishing the diagnosis.

**Methods:**

Patients who presented with apparent primary spontaneous pneumothorax between 2004 and 2017 in a large Dutch teaching hospital were enrolled in this quantitative cross-sectional study. A questionnaire was sent to eligible patients. Patients who completed the questionnaire and consented to further participation were invited to visit the hospital for genetic testing and low dose, volumetric chest CT.

**Results:**

Genetic testing was performed in 88 patients with apparent primary spontaneous pneumothorax. Three patients were found to have a pathogenic variant in the *FLCN* gene (3.4%). No variants of unknown significance were detected. Pulmonary cysts were detected in 14 out of 83 participants with an available chest CT, six had more than one cyst. All three patients with BHD had multiple pulmonary cysts.

**Conclusions:**

Based on previous literature and the present study, we believe that performing a chest CT in every patient presenting with primary spontaneous pneumothorax is justified. Subsequent genetic testing of the FLCN gene should be considered when multiple pulmonary cysts are present.

***Trial registration*:**

The study was registered at clinicaltrials.gov with reference NCT02916992.

**Summary at a glance:**

Three out of 88 patients with an apparent primary spontaneous pneumothorax were diagnosed with Birt-Hogg-Dubé syndrome in this study and all three had multiple pulmonary cysts. We believe that performing a chest CT in every patient with an apparent primary spontaneous pneumothorax is justified to identify underlying diseases.

## Background

Birt-Hogg-Dubé syndrome (BHD) is an autosomal dominant condition caused by loss of function mutations in the *FLCN* gene [1–3]. It is characterized by fibrofolliculomas in 49–84% of the patients, pulmonary cysts in 70–89%, pneumothorax in 24–38% and renal cell carcinoma (RCC) in 14–34% [4–12]. Given the variable expression, BHD is probably underdiagnosed [13]. Symptoms of RCC often develop in late stages of the disease and early detection gives the opportunity to treat tumors at an early stage, which can be lifesaving [14–16]. A diagnosis of BHD allows for renal surveillance in both patients and their relatives carrying the *FLCN* pathogenic variant.

A possible way to identify more BHD patients at risk for RCC is to test for BHD among patients presenting with apparent primary spontaneous pneumothorax (PSP). Guidelines on PSP specify that chest computed tomography (CT) is seldom indicated considering the low added diagnostic value and several guidelines do not even mention CT [17–19]. As a result, pulmonary cysts, which could be an indication of BHD, are often not recognized. The cysts in BHD are typically well circumscribed, irregularly shaped, located in the basal parts of the lungs, vary in size, are relatively often attached to the pleura and they tend to be associated with blood vessels [20, 21]. Other features of BHD, such as skin lesions and a family history of RCC or pneumothorax, are not always routinely assessed by most pulmonologists. Therefore, the diagnosis of BHD may easily be missed. It is therefore relevant to assess the prevalence of BHD among patients with apparent PSP and to determine whether chest CT and/or genetic testing in all PSP patients is justified. The prevalence of BHD among patients with familial pneumothorax has been reported to be 17–86% [22–24]. The broad range is probably due to a difference in inclusion criteria and a small number of families per study. The prevalence of BHD in unselected patients with apparent PSP has been investigated in three previous studies. First, Johannesma et al. have performed a retrospective study in 40 apparent PSP patients in another Dutch hospital, and BHD was diagnosed in 7.5% of the patients [25]. Furthermore, 102 patients with apparent PSP have been analyzed in a Chinese study and BHD was detected in 9.8% [26]. Finally, Zhang et al. have investigated a NGS strategy for diagnosing BHD, as well as other inherited diseases with an increased risk for pneumothorax, in patients with apparent PSP. Two out of 21 patients carried a pathogenic variant and one a likely pathogenic variant in the *FLCN* gene [27]. Ebana et al. studied patients with pneumothorax who underwent VATS during a given time period. In their study, 9.5% percent of patients with PSP undergoing VATS had a known diagnosis of BHD [28].

In this study, we aimed to examine the prevalence of BHD in patients presenting with PSP by genetic testing. Also, we aimed to evaluate the contribution of CT in diagnosing BHD in PSP patients and to provide information on the characteristics of cysts in all patients with pulmonary cysts.

## Methods

### Study population

This quantitative cross-sectional study was performed in the Rijnstate Hospital in Arnhem, The Netherlands. The study was approved by the ethical committee of Radboud University Medical Center in Nijmegen with approval number 2014–525. The study is registered at clinicaltrials.gov with reference NCT02916992 at 28/09/2016.

Written informed consent was obtained from all included patients. The medical records of all patients who presented with a pneumothorax between 2004 and 2017 were assessed and considered for inclusion if no cause of the pneumothorax was mentioned in the hospital discharge letter. Exclusion criteria were secondary, traumatic or iatrogenic pneumothorax, age below 18, insufficient proficiency in the Dutch language and intellectual disability. A letter and a questionnaire were sent to the home addresses of patients meeting the inclusion criteria. Patients who completed the questionnaire and consented to further participation in research, received more information about the study and were invited for a hospital visit for skin assessment, genetic testing and chest CT.

### Questionnaire

The questionnaire consisted of questions regarding the number, date and sides of pneumothoraces, patient history, comorbidity, smoking habits and familial history.

### Genetic testing

*FLCN* testing was performed in the diagnostic DNA laboratory of the VU University Medical Center in Amsterdam. The analysis was carried out on DNA extracted from a venous blood sample and performed by Sanger sequencing and multiplex ligation-dependent probe amplification (MLPA) as previously described [8]. Residual material was destroyed unless patients consented with further use in scientific research.

### Computed tomography

A chest CT for the purpose of this study was only performed if no suitable previous chest CT of the patient was available for the assessment of cysts (e.g. chest CT without a large pneumothorax). As a result, not all CT scans assessed were performed using the same type of scanner. The low dose volumetric chest CT scans for the purpose of this study were performed using the Somatom Emotion 16 (Siemens, München, Germany). If CT scans of the patient were already available, the last suitable scan was assessed for cysts. The smallest slice thickness ranged between 0.75 and 4.00 mm. The CT scans were assessed for emphysema-like changes, cysts and additional relevant findings by the radiologist and the pulmonologist. For all cysts, the number, the association with blood vessels, the location, the size and the shape were assessed. In case of incidental findings on the chest CT, the patient was informed and referred to a specialist for further evaluation if indicated.

### Statistical analysis

The statistical analyses were performed using SPSS version 25.0 (IBM Corp. Released 2017. IBM SPSS Statistics for Windows, Version 25.0. Armonk, NY: IBM Corp). Descriptive statistics were used to display demographic data. The Fisher’s exact test was used to compare dichotomous variables between cysts in patients with and without BHD.

## Results

### Population

**Fig. 1:**
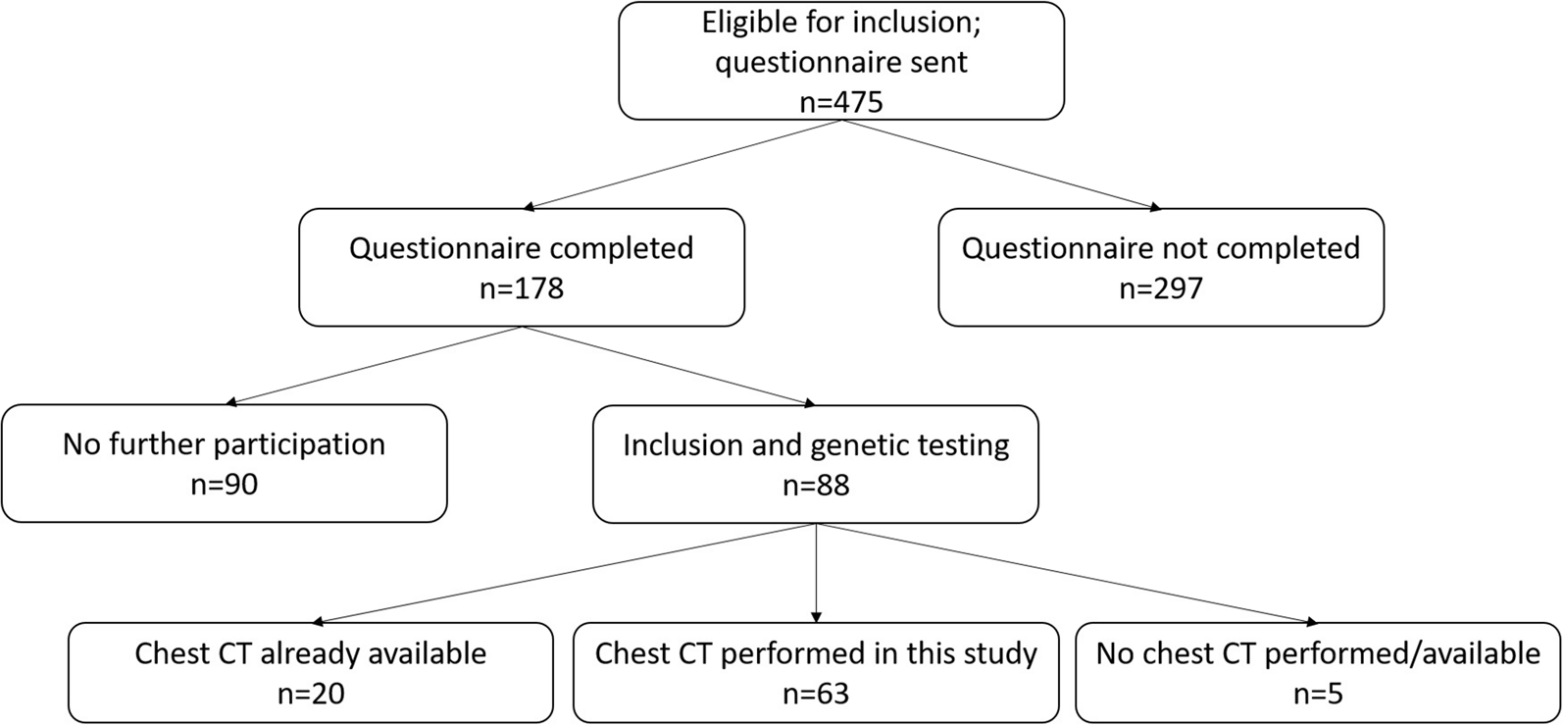
Study flowchart

475 apparent spontaneous pneumothorax patients met the inclusion criteria and were sent a questionnaire. A total of 178 patients completed and returned the questionnaire and these patients received further information about the study. Among them, 90 refused to participate, had relocated, or their pneumothorax was found to be secondary at further inspection of the medical files. Ultimately, 88 patients were included for genetic testing. In 20 of them, a chest CT for the evaluation of cysts was already available. A total of 63 patients underwent a chest CT for the purpose of this study; 44 never had a chest CT before and 19 had a chest CT during pneumothorax which was not eligible to asses for cysts. Five patients refrained from a chest CT after all and only had genetic testing performed. Figure 1 shows a flow chart of the patient selection. The demographic characteristics and questionnaire results are shown in Table 1.

**Table 1:** Demographic characteristics and results of chest CT and genetic testing

	All included patients (n = 88)	Chest CT already available (n = 20)	Chest CT performed in this study (n = 63)	No chest CT (n = 5)
Age in years, median (range)	34 (18–74)	36 (19–74)	35 (18–66)	22 (21–34)
Female	36 (40.9)	10 (50.0)	24 (38.1)	2 (40.0)
Number of pneumothoraxes, median (range)	1 (1–7)	2 (1–6)	1 (1–7)	1 (1–1)
Age at first pneumothorax in years, median (range)	29 (16–74)	28.5 (17–74)	30 (16–58)	18 (17–25)
Side of pneumothorax				
Left	34 (38.6)	5 (25.0)	28 (44.4)	1 (20.0)
Right	45 (51.1)	11 (55.0)	30 (47.6)	4 (80.0)
Bilateral	9 (10.2)	4 (20.0)	5 (7.9)	0
Family history of pneumothorax	19 (21.6)	4 (20.0)	14 (22.2)	1 (20.0)
Family history of renal cell carcinoma	5 (5.7)	3 (15.0)	2 (3.2)	0
Smoking				
Current smoker	27 (30.7)	5 (20.0)	20 (31.7)	2 (40.0)
Past smoker	39 (44.3)	11 (55.0)	27 (42.9)	1 (20.0)
Never smoked	22 (25.0)	4 (20.0)	16 (25.4)	2 (40.0)
Chest CT	(out of n = 83)			
1 cyst	8 (9.6)	3 (15.0)	5 (7.9)	NA
> 1 cyst	6 (7.2)	4 (20.0)	2 (3.2)	NA
Emphysema-like changes	35 (42.2)	9 (45.0)	26 (41.3)	NA
*FLCN* pathogenic variant	3 (3.4)	2 (10.0)	1 (1.6)	0

Results are presented as n(%) unless otherwise stated

*NA* Not applicable

### Genetic testing

Two pathogenic variants in *FLCN* were detected in three patients (3.4%): c.1301-7_1304del; 1323delinsGA (twice), and c.1408_1418del. No variants of unknown significance were detected. Both pathogenic variants have been reported previously in multiple BHD patients [8, 25, 29]. The patient characteristics are shown in Table 2. All three patients were female and had skin lesions suspect of fibrofolliculomas. Patient 1 and 2 were mother and daughter who had 7 more family members with a history of pneumothorax.

**Table 2:** Characteristics of BHD patients

	BHD patient 1	BHD patient 2	BHD patient 3
Age	51	26	38
Gender	Female	Female	Female
Number of pneumothoraxes	1	1	6
Age at first pneumothorax in years	40	22	21
Side of pneumothorax	Right	Right	Bilateral
Family history of pneumothorax	Yes (in 8 family members)	Yes (in 8 family members)	No
Family history of renal cell carcinoma	No	No	Yes (maternal aunt)
Smoking	Past smoker	Past smoker	Current smoker
Skin examination	Suspect for fibrofolliculomas	Suspect for fibrofolliculomas	Suspect for fibrofolliculomas
Moment of chest CT	In this study	Already available	Already available
Result of chest CT	3 cysts	3 cysts	9 cysts Emphysema
*FLCN* pathogenic variant	c.1301-7_1304del; 1323delinsGA	c.1301-7_1304del; 1323delinsGA	c.1408_1418del

### Pulmonary cysts

**Fig. 2:**
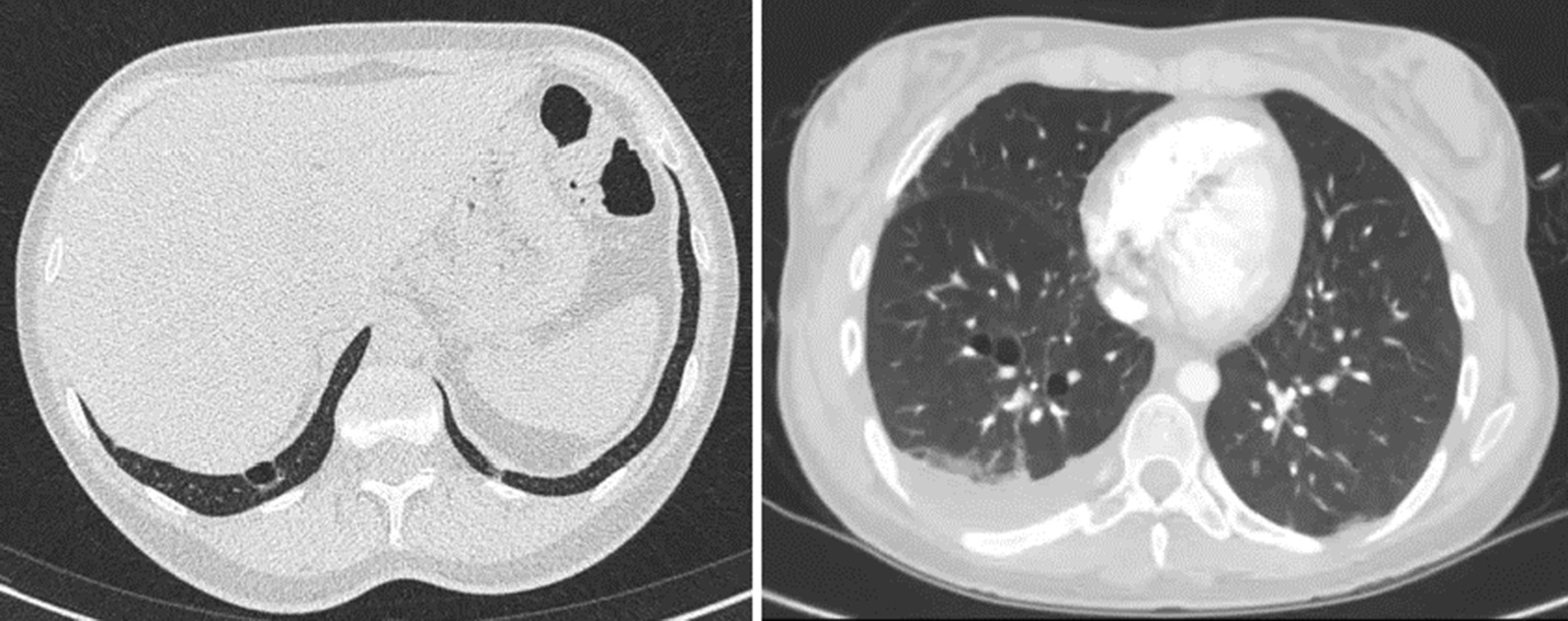
Pulmonary cysts in two patients with BHD

A chest CT was available from a total of 83 patients. Cysts were present in fourteen patients of whom six patients had multiple cysts. Three patients without a *FLCN* pathogenic variant had two cysts each and were not suspected to have another underlying lung disease. All patients with BHD had multiple cysts (n = 3, 3 and 9 respectively). The characteristics of the cysts are shown in Table 3. The majority of cysts were located in the basal lung fields below the level of the carina. The cysts in the patients with BHD were significantly more often associated with a blood vessel. Examples of cysts of 2 of the patients with BHD are shown in Fig. 2.

**Table 3:** Characteristics of pulmonary cysts

	BHD (n = 15 cysts from 3 patients)	non-BHD (n = 14 cysts from 11 patients)	p-value
Association with blood vessel	13 (86.7)	4 (28.6)	0.003
Parenchymal location	10 (66.7)	5 (35.7)	0.14
Located under the carina	12	12	1
Size > 1 cm	7 (46.7)	6 (42.9)	1
Irregular shape	5 (33.3)	4 (28.6)	1

Results are presented as n (%)

## Discussion

In this study, we aimed to determine the prevalence of BHD among patients presenting with apparent PSP. Furthermore, CT scans of patients with apparent PSP were assessed on pulmonary cysts to evaluate the contribution of chest CT in diagnosing patients with BHD. The general characteristics of included individuals show, as expected, a higher percentage of smokers than in the general population, a high prevalence of emphysema-like changes on their chest CT and a higher percentage of males. All are known risk factors for pneumothorax [30–32].

We identified a pathogenic variant in *FLCN* in three out of 88 apparent PSP patients (3.4%). Previous studies reported a higher prevalence of 7.5–14.3% [25–28]. A possible explanation for the difference might be the relative small sample size in our study and other reported studies. Differences between the studies concerning the inclusion criteria or characteristics of included patients may also have played a role. However, other studies did not have more patients with a family history for pneumothorax (21.6% in our study, 9.8% in Ren et al. [26]). Not all studies reported the timing and results of chest CT, which we could therefore not compare. Lastly, we hypothesize that the discrepancy in prevalence could partly be attributed to a difference between the Dutch and Chinese populations. Tall stature is a risk factor for PSP [33], and Dutch people are among the tallest people in the world [34]. The studies of Ren et al. and Zhang et al. [26, 27] were conducted in China, where the prevalence of pneumothorax in the general population might be lower than that in the Netherlands, resulting in relatively more patients with underlying conditions such as BHD. This hypothesis is still speculative because the exact prevalence of PSP in both countries is unknown and it is also not clear how the length of BHD patients influences their pneumothorax risk. Although the yield in our study was lower than previously described, we did identify two new families with BHD. This led to the diagnosis of a hybrid oncocytic-chromophobe renal carcinoma with a maximum diameter of 2.6 cm in one of the participating patients. It was successfully removed by partial nephrectomy. At-risk family members from both families were informed about BHD and were given the possibility to perform genetic testing for BHD and undergo renal surveillance if the *FLCN* pathogenic variant was detected.

The three BHD patients in our study in retrospect all had recognizable features of BHD that had could have been an indication for genetic testing at an earlier time. Patients 1 and 2 were a mother and daughter from a family with many family members with pneumothorax. The daughter already had a CT-scan available in her medical records that showed pulmonary cysts. Patient 3 also had a CT-scan available that showed multiple cysts and she had a family history of RCC. Furthermore, all three patients had skin lesions suspect of fibrofolliculomas. This suggests that raising awareness of BHD among pulmonologists and radiologists can be an important factor in identifying more families with BHD. BHD should be considered as an underlying cause in patients with apparent spontaneous pneumothorax who have skin lesions or report a family history of RCC or PSP, especially if a chest CT has not been performed. In addition, genetic testing for BHD is indicated in all patients with multiple pulmonary cysts without a known cause.

Although pulmonary cysts are not fully penetrant in BHD, they were present in all three BHD patients in this study and in all patients the previous independent Dutch study [25]. In the current study, the yield of pathogenic *FLCN* variants in individuals with pulmonary cysts was 3/14 (21.4%) and among individuals with multiple pulmonary cysts 3/6 (50.0%). This is more or less comparable to a previous study where in 6/17 (35.3%) individuals with a chest CT suspect for BHD, the diagnosis of BHD was confirmed [35]. Other researchers proposed a scoring system to select patients with PSP who should undergo genetic testing for BHD [28]. The items of the proposed score system were a family history of pneumothorax (3 points), history of bilateral pneumothorax (3 points), age of 25 years or older at the first episode of pneumothorax (2 points), being female (2 points) and a body mass index of 18.5 or higher (1 point). The cut-off point was a score of at least four. In the current study, we only have data about the first four features. Nevertheless, all three BHD patients in our study would have had a score above the cut-off point (at least: 5, 5 and 7). Among the individuals without BHD, at least 33/85 also had a score of four or higher, leading to a sensitivity of 100% and a specificity of 54–61% of this scoring system in our study population. Another recent study showed cost-effectiveness of high-resolution CT (HRCT) chest imaging in diagnosing cystic lung diseases, such as BHD and lyphangioleiomyomatosis, in patients with apparent PSP [36]. A chest CT may also help to diagnose other lung diseases such as emphysema, which was present in over 50% of our study population, and alpha-1-antitrypsin deficiency. These diagnoses can have implications for treatment and/or might be a motivation for patients to quit smoking.

Cysts in BHD have been reported to be more frequently associated with a blood vessel and more often parenchymal compared to cysts in pneumothorax patients without BHD [20, 37]. This was also observed in the present study. Furthermore, cysts in BHD have been reported to be predominantly irregularly shaped and larger than one centimeter [20, 21, 38]. We did not observe these two characteristics. This might be due to the small number of cysts that were evaluated in the present and previous studies. Additionally, this might also reflect the fact that there is heterogeneity in the presentation of pulmonary cysts in BHD and establishing a diagnosis based on chest CT solely is not possible.

This study has some limitations. The response rate to the questionnaire (37,5%) was lower than expected. There was no significant difference in age between the responders and non-responders though (data not shown). The questionnaire did not mention BHD, so a selection bias towards patients with a positive personal or family history for features of BHD cannot have played a role at the questionnaire stage. Only half of the responders was included for genetic testing, which might have caused a selection bias. After completing the questionnaire, patients were informed about the aim of the study and patients with a positive (family) history of features of BHD might have been more prone to participate in the study.

## Conclusions

BHD was the cause of the pneumothorax in 3.4% of patients with apparent PSP. Performing a chest CT in every patient presenting with PSP is justified based on previous literature and the present study. Subsequent genetic testing for BHD should be advised in case pulmonary cysts are present. Our results show that currently not all patients with pulmonary cysts are tested for BHD and raising awareness about this matter is important to detect families with BHD. The goal of the chest CT would be to identify more patients with BHD, which allows for genetic testing in family members and renal surveillance for those with BHD. Also, performing a chest CT may lead to the detection of other relevant lung diseases.

## Data Availability

The datasets generated during the current study are available from the corresponding author on reasonable request.

## Abbreviations

BHD: Birt-Hogg-Dubé syndrome
CT: Computed tomography
PSP: Primary spontaneous pneumothorax
RCC: Renal cell carcinoma

## References

[1] Khoo SK, Bradley M, Wong FK, Hedblad MA, Nordenskjold M, Teh BT (2001). Birt-Hogg-Dube syndrome: mapping of a novel hereditary neoplasia gene to chromosome 17p12-q11.2. Oncogene.

[2] Schmidt LS, Warren MB, Nickerson ML, Weirich G, Matrosova V, Toro JR (2001). Birt-Hogg-Dube syndrome, a genodermatosis associated with spontaneous pneumothorax and kidney neoplasia, maps to chromosome 17p11.2. Am J Hum Genet.

[3] Nickerson ML, Warren MB, Toro JR, Matrosova V, Glenn G, Turner ML (2002). Mutations in a novel gene lead to kidney tumors, lung wall defects, and benign tumors of the hair follicle in patients with the Birt-Hogg-Dube syndrome. Cancer Cell.

[4] Birt AR, Hogg GR, Dube WJ (1977). Hereditary multiple fibrofolliculomas with trichodiscomas and acrochordons. Arch Dermatol.

[5] Toro JR, Glenn G, Duray P, Darling T, Weirich G, Zbar B (1999). Birt-Hogg-Dube syndrome: a novel marker of kidney neoplasia. Arch Dermatol.

[6] Zbar B, Alvord WG, Glenn G, Turner M, Pavlovich CP, Schmidt L (2002). Risk of renal and colonic neoplasms and spontaneous pneumothorax in the Birt-Hogg-Dube syndrome. Cancer Epidemiol Biomark Prev.

[7] Furuya M, Yao M, Tanaka R, Nagashima Y, Kuroda N, Hasumi H (2016). Genetic, epidemiologic and clinicopathologic studies of Japanese Asian patients with Birt-Hogg-Dube syndrome. Clin Genet.

[8] Houweling AC, Gijezen LM, Jonker MA, van Doorn MB, Oldenburg RA, van Spaendonck-Zwarts KY (2011). Renal cancer and pneumothorax risk in Birt-Hogg-Dube syndrome; an analysis of 115 FLCN mutation carriers from 35 BHD families. Br J Cancer.

[9] Schmidt LS, Nickerson ML, Warren MB, Glenn GM, Toro JR, Merino MJ (2005). Germline BHD-mutation spectrum and phenotype analysis of a large cohort of families with Birt-Hogg-Dube syndrome. Am J Hum Genet.

[10] Toro JR, Wei MH, Glenn GM, Weinreich M, Toure O, Vocke C (2008). BHD mutations, clinical and molecular genetic investigations of Birt-Hogg-Dube syndrome: a new series of 50 families and a review of published reports. J Med Genet.

[11] Toro JR, Pautler SE, Stewart L, Glenn GM, Weinreich M, Toure O (2007). Lung cysts, spontaneous pneumothorax, and genetic associations in 89 families with Birt-Hogg-Dube syndrome. Am J Respir Crit Care Med.

[12] Kluger N, Giraud S, Coupier I, Avril MF, Dereure O, Guillot B (2010). Birt-Hogg-Dube syndrome: clinical and genetic studies of 10 French families. Br J Dermatol.

[13] Steinlein OK, Ertl-Wagner B, Ruzicka T, Sattler EC (2018). Birt-Hogg-Dube syndrome: an underdiagnosed genetic tumor syndrome. J Dtsch Dermatol Ges.

[14] Schips L, Lipsky K, Zigeuner R, Salfellner M, Winkler S, Langner C (2003). Impact of tumor-associated symptoms on the prognosis of patients with renal cell carcinoma: a single-center experience of 683 patients. Urology.

[15] Low G, Huang G, Fu W, Moloo Z, Girgis S (2016). Review of renal cell carcinoma and its common subtypes in radiology. World J Radiol.

[16] Sunela KL, Kataja MJ, Kellokumpu-Lehtinen PL (2010). Changes in symptoms of renal cell carcinoma over four decades. BJU Int.

[17] Baumann MH, Strange C, Heffner JE, Light R, Kirby TJ, Klein J (2001). Management of spontaneous pneumothorax: an American college of chest physicians delphi consensus statement. Chest.

[18] MacDuff A, Arnold A, Harvey J, Group BTSPDG (2010). Management of spontaneous pneumothorax: British thoracic society pleural disease guideline 2010. Thorax.

[19] Schramel FMNH AJ, Mannes GPM, Smit JM, Willems LNA. Primaire spontane pneumothorax: richtlijn van de Nederlandse Vereniging van Artsen voor Longziekten en Tuberculose. Alphen aan den Rijn, Van Zuiden Communications BV 2002.

[20] Park HJ, Chae EJ, Do KH, Lee SM, Song JW (2019). Differentiation between lymphangioleiomyomatosis and Birt-Hogg-Dube syndrome: analysis of pulmonary cysts on CT images. AJR Am J Roentgenol.

[21] Tobino K, Gunji Y, Kurihara M, Kunogi M, Koike K, Tomiyama N (2011). Characteristics of pulmonary cysts in Birt-Hogg-Dube syndrome: thin-section CT findings of the chest in 12 patients. Eur J Radiol.

[22] Graham RB, Nolasco M, Peterlin B, Garcia CK (2005). Nonsense mutations in folliculin presenting as isolated familial spontaneous pneumothorax in adults. Am J Respir Crit Care Med.

[23] Torricelli E, Occhipinti M, Cavigli E, Tancredi G, Rosi E, Rossi C (2019). The relevance of family history taking in the detection and management of Birt-Hogg-Dube syndrome. Respiration.

[24] Liu Y, Xing H, Huang Y, Meng S, Wang J (2019). Familial spontaneous pneumothorax: importance of screening for Birt-Hogg-Dube syndrome. Eur J Cardiothorac Surg.

[25] Johannesma PC, Reinhard R, Kon Y, Sriram JD, Smit HJ, van Moorselaar RJ (2015). Prevalence of Birt-Hogg-Dube syndrome in patients with apparently primary spontaneous pneumothorax. Eur Respir J.

[26] Ren HZ, Zhu CC, Yang C, Chen SL, Xie J, Hou YY (2008). Mutation analysis of the FLCN gene in Chinese patients with sporadic and familial isolated primary spontaneous pneumothorax. Clin Genet.

[27] Zhang X, Ma D, Zou W, Ding Y, Zhu C, Min H (2016). A rapid NGS strategy for comprehensive molecular diagnosis of Birt-Hogg-Dube syndrome in patients with primary spontaneous pneumothorax. Respir Res.

[28] Ebana H, Mizobuchi T, Kurihara M, Kobayashi E, Haga T, Okamoto S (2018). Novel clinical scoring system to identify patients with pneumothorax with suspicion for Birt-Hogg-Dube syndrome. Respirology.

[29] Kluijt I, de Jong D, Teertstra HJ, Axwijk PH, Gille JJ, Bell K (2009). Early onset of renal cancer in a family with Birt-Hogg-Dube syndrome. Clin Genet.

[30] Springvloet L BJ, Willemsen M, van Laar M. Kerncijfers roken 2017. Trimbos-instituut. 2018.

[31] Bense L, Eklund G, Wiman LG (1987). Smoking and the increased risk of contracting spontaneous pneumothorax. Chest.

[32] Bobbio A, Dechartres A, Bouam S, Damotte D, Rabbat A, Regnard JF (2015). Epidemiology of spontaneous pneumothorax: gender-related differences. Thorax.

[33] Melton LJ, Hepper NG, Offord KP (1981). Influence of height on the risk of spontaneous pneumothorax. Mayo Clin Proc.

[34] Collaboration NCDRF (2016). A century of trends in adult human height. J Elife.

[35] Park HJ, Park CH, Lee SE, Lee GD, Byun MK, Lee S (2017). Birt-Hogg-Dube syndrome prospectively detected by review of chest computed tomography scans. PLoS One.

[36] Gupta N, Langenderfer D, McCormack FX, Schauer DP, Eckman MH (2017). Chest computed tomographic image screening for cystic lung diseases in patients with spontaneous pneumothorax is cost effective. Ann Am Thorac Soc.

[37] Kumasaka T, Hayashi T, Mitani K, Kataoka H, Kikkawa M, Tobino K (2014). Characterization of pulmonary cysts in Birt-Hogg-Dube syndrome: histopathological and morphometric analysis of 229 pulmonary cysts from 50 unrelated patients. Histopathology.

[38] Agarwal PP, Gross BH, Holloway BJ, Seely J, Stark P, Kazerooni EA (2011). Thoracic CT findings in Birt-Hogg-Dube syndrome. AJR Am J Roentgenol.

